# Calibration of AI large language models with human subject matter experts for grading of clinical short-answer responses in dental education

**DOI:** 10.1186/s12903-026-07665-4

**Published:** 2026-02-06

**Authors:** Fatma E.A. Hassanein, Radwa R. Hussein, Yousra Ahmed, Jylan El-Guindy, Doha E. Ahmed, Asmaa Abou-Bakr

**Affiliations:** 1 Faculty of Dentistry, Oral Medicine, Periodontology, and Oral Diagnosis, King Salman International University, El-Tor, Egypt; 2 Oral Medicine and Periodontology, Ain Shams University in Egypt, Cairo, Egypt; 3 Faculty of Dentistry, Prosthodontics Dentistry, King Salman International University, El-Tor, Egypt; 4 Clinical and Chemical Pathology Department, Faculty of Medicine, Misr University for Sciences and Technology, Giza, Egypt; 5 Faculty of Dentistry, Oral Medicine and Periodontology, Galala University, Suez, Egypt

**Keywords:** Artificial intelligence, Automated assessment, Short-answer grading, Large language models, LLM, Dental education, Dental examination, Rubric scoring

## Abstract

**Background:**

The automated grading of clinical short-answer questions using large language models (LLMs) could alleviate faculty workload and improve the immediacy of feedback in dental education. However, evidence on the capacity of LLMs for rubric-based grading in dentistry remains limited. Therefore, this study aimed to compare the grading reliability and error patterns of two LLMs, ChatGPT-4 and the open-weight DeepSeek-3, against expert human evaluators.

**Materials and methods:**

In a retrospective cross-sectional study with comparative validation design, we analyzed 2,358 short-answer responses from 262 undergraduate dental students (across nine clinical questions). All responses were analyzed, then human-graded by three calibrated subject-matter experts (SME) (intraclass correlation coefficient [ICC] = 0.84) to provide a reference. Each LLM was provided a 12-point analytic rubric to guide the grading, but was not provided any prior examples of the grading task (i.e., a zero-shot prompt). We assessed agreement using ICC, Pearson correlation, Cohen’s kappa, and mixed-effects models, and examined error tiers (≤ 1, 2–3, > 3 points) across Bloom’s levels and response styles.

**Results:**

In this dataset, DeepSeek-3 obtained an ICC of 0.87 compared with ChatGPT-4 which obtained an ICC of 0.64. DeepSeek-3 matched exactly with human scores in 43.3% of cases and was within ± 1 point in 62.4%, compared with 35.5% and 44.1% for ChatGPT-4. High-error rates (> 3 points) were 7.5% for DeepSeek-3 vs. 26.9% for ChatGPT-4 (χ², *p* < 0.01). DeepSeek-3’s agreement was consistent across cognitive levels and response verbosity, while ChatGPT-4’s accuracy on higher-level and verbose responses was significantly lower (*p* < 0.01). Both models exhibited an optimistic bias by over-scoring incorrect answers.

**Conclusions:**

DeepSeek-3 showed fewer large-magnitude errors and better agreement with human graders compared to ChatGPT-4, suggesting its potential value for large-scale AI-assisted assessment for dental education. Since both models can over-score on incorrect results, human-in-the-loop oversight is necessary for high-stakes applications. Further work should evaluate performance across more courses, institutions, and languages, as well as examine the effects of model calibration, bias reduction, and external validation before considering the broader integration of LLMs into dental education.

**Supplementary Information:**

The online version contains supplementary material available at 10.1186/s12903-026-07665-4.

## Introduction

The increasing use of AI tools in education for feedback and grading has raised concerns about whether these systems can match the judgment of human assessors. This issue is especially pertinent in clinical education, where competence involves more than simple knowledge recall. The application of large language models (LLMs) for health education thus comes with opportunities and challenges. Despite their high scalability, how well they match human scoring, particularly for higher-order cognitive skills, has received little attention [1, 2].

Additionally, applying AI-assisted grading to dental education poses unique challenges [3]. Unlike multiple-choice exercises, open-ended responses also require evaluators, human or AI, to interpret clinical context, scientific reasoning, and use of domain-dependent terminology. These observations make rubric adherence more challenging and call for models that can accommodate diverse student expression well. This highlights the importance of the direct comparison of commercial and open-access LLMs in standardized, real-world evaluation environments, which is the goal of the work presented in this paper [4–7].

Short-answer (SA) and case-based questions are increasingly preferred in dental education due to their capability to measure clinical reasoning and integration of thinking more authentically than multiple-choice items. These assessments suffer from practical challenges: mid-size cohorts can yield thousands of individual responses, which need to be double-marked by calibrated graders for consistency, timeliness of feedback, and to avoid concerns about inter-rater agreement and measurement error [8]. The Association for Dental Education in Europe suggests in their standards that authenticity and feasibility should be balanced against each other and that assessor inconsistency should be reduced [9]. Large language models (LLMs), such as OpenAI’s GPT-4 and its variants, have become a scalable approach for simulating expert reasoning [10–12], generating educational content, and assessment, as well as evaluating language modeling with near-human fluency. Nowadays, in education, they are used as both feedback engines and virtual tutors, and for automatic grading [13].

Medical education studies initially focused on kappa coefficients and presented evidence of moderate agreement between ChatGPT-generated grades and human ratings across different SA questions (κ ≈ 0.60–0.70), cautioning about rubric sensitivity and error variation [2, 14]. One such large-scale analysis of 2,288 student responses validated GPT-4’s sufficient grading accuracy in combination with adequate safeguarding in the form of outlier detection and infrequent human review [6].

Despite such progress, the available studies on the use of AI in dental education are limited. The majority of studies focus on the performance of LLMs in board-style multiple-choice questions or creating tests, rather than assessing LLMs’ free-text clinical responses [15]. In addition, certain models’ potential to grade in stratified conditions has not been adequately evaluated, such as varying levels of respondent quality or cognitive complexity. One significant area of concern is the efficacy of open-weight LLMs, such as DeepSeek-V3, a configurable on-premises model with competitive benchmark performance but insufficient empirical validation in health education situations. Previous research has shown that these models may not perform well for tasks that require more sophisticated reasoning or domain-specific rubric interpretation [16].

Previous studies have assessed chatbot proficiency on dental board exams and accuracy in responding to specialist clinical questions, such as pediatric dentistry, special needs dentistry, and prosthodontics [17–20]. Although such studies help shape the factual knowledge and clinical decision-making of LLMs, they have primarily focused on multiple-choice question types or the correctness of AI-generated answers in themselves. There is relatively less evidence regarding the consistency of such models in performing a core pedagogical function: machine-driven, rubric-aided grading of student-generated clinical short-answer responses.

While ChatGPT-4 is a proprietary model trained on a broad, heterogeneous body of written texts and widely recognized for its strong performance across general and medical natural language tasks [21]. Studies have noted its variable reliability and occasional misalignment with expert rubric standards in clinical education, especially in open-ended or nuanced scenarios [6, 14]. In contrast, DeepSeek-3 is an open-weight model with openly accessible internal parameters and core architecture, designed for on-premises deployment and trained on technical and instructional subject matter, which may enhance its capacity for rule-following and domain-specific rubric alignment [4]. However, unlike ChatGPT-4, DeepSeek-3 has not been widely validated in clinical or health education contexts, and its generalizability and grading accuracy in these settings remain untested. These architectural and data differences suggest the potential for divergent strengths and limitations in real-world educational assessment tasks, making direct comparison essential.

This investigation makes a unique contribution to the dental education literature by comparing commercial and open-weight LLMs in grading genuine, rubric-scored clinical responses against one another. By examining the reliability, rubric drift, cognitive stratification, and bias of the models, this study offers empirically grounded practical guidance on the application of AI to high-stakes dental assessments.

This study aimed to address this gap in the literature by comparing two of the most prominent LLMs (OpenAI’s ChatGPT-4 and the open-weight DeepSeek-3) in the context of rubric-scored, short-answer clinical questions in undergraduate dental education. The primary objective was to determine the reliability and accuracy of each model compared with a calibrated human grader. The secondary objective was to describe model-specific error patterns and investigate whether disagreements varied by level of rubric, cognitive level, response format, or model form, thus providing a nuanced portrayal of AI–human alignments in dental performance assessment. Given DeepSeek-3’s domain alignment and prior evidence of GPT-4’s variability, we hypothesized that DeepSeek-3 would exhibit better grading reliability and fewer error rates than ChatGPT-4 due to its compatibility with technical instruction and rule-following tasks.

## Methods

### Study design and timeline

This was a cross-sectional, retrospective observational study during the 2024–2025 academic year at the Faculty of Dentistry, Ain Shams University, Cairo (Egypt). Reporting follows the STROBE checklist for cross-sectional investigations.

### Ethical considerations

While the responses analyzed in this study were derived from students’ mandatory, summative end-of-course examination, these responses were anonymized for research purposes, and no direct impact on the students’ actual course grades occurred as a result of their participation in the study. Ethical approval for the study was obtained, and a waiver of informed consent was granted by the Research Ethics Committee of the Faculty of Dentistry, Ain Shams University (Approval No. FDASU-RecER072508). The research procedures complied with institutional data protection policies and maintained the strict confidentiality of student information. The Declaration of Helsinki’s ethical guidelines were followed when conducting the study.

### Outcomes measure

The aim was to compare the grading performance of two large-language models (LLMs), ChatGPT-4 (OpenAI, 2025 API) and DeepSeek-3 (2025 public release), with that of calibrated human subject-matter experts (SMEs) on rubric-scored, short-answer clinical questions.

### Participants and data set

The study focused solely on the analysis of the students’ exam responses from a cohort of 262 undergraduate dental students enrolled in the Periodontology course, which did not affect their official grades. The mandatory, summative end-of-course examination contained nine open-ended clinical questions, generating 2,358 short-answer responses (262 students × 9 questions) as displayed in the Supplementary File 1.

A priori power analysis for inter-rater reliability (expected ICC ≈ 0.70; null = 0.40; α = 0.05; power = 0.80) indicated that ≥ 60 responses would suffice, so the available sample was more than adequate for all planned analyses.

Students completed the short-answer examination via the faculty’s Moodle learning management system (LMS); responses were exported digitally as text data for grading.

### Assessment structure and cognitive stratification

Each item required application of clinical reasoning (diagnosis, interpretation, or management). By expert consensus, three questions were mapped to each Bloom’s cognitive taxonomy level: Recall/Comprehension, Application/Analysis, and Evaluation/Synthesis, ensuring a balanced cognitive spectrum (33.3% per level).

For illustration, a representative item at the Recall/Comprehension level, ***“****Oral biofilms play a dual role in oral health and disease.* *Question*: *Describe the main functions of oral biofilm in both health and disease.”*

At the Application/Analysis level, ***“****Not all periodontal defects are suitable for regenerative therapy.* *Question*: *List five key clinical and radiographic criteria for determining if a periodontal defect is suitable for regenerative therapy.”*

At the Evaluation/Synthesis level, *“42-year-old male presents with a narrow*,* deep*,* three-wall intrabony defect on the mesial aspect of tooth #12*,* thin gingival phenotype*,* intact papilla*,* non-smoker*,* controlled hypertension. He requests a surgical option with minimal postoperative discomfort and downtime.* *Question*: *Choose the most appropriate initial surgical technique and explain why it is indicated over alternatives.”*

### Rubric development

An analytic 12-point scoring rubric was constructed by faculty consensus to evaluate four performance domains: Clarity, Accuracy, Completeness, and Grammar & Language Use, each consisting of three items scored from 0 (poor) to 3 (excellent). Internal-consistency testing on pilot responses demonstrated strong reliability, with Cronbach’s alpha values of 0.82 for Clarity, 0.85 for Accuracy, 0.80 for Completeness, and 0.87 for Grammar & Language Use; the full 12-item scale achieved an overall alpha of 0.84. Pilot application to sample answers prompted only minor wording refinements before the rubric was deployed in the main study.

### Human grading procedure

Three trained SMEs independently scored all 2,358 responses while blinded to one another and to AI outputs. After a calibration workshop, the rubric was applied individually; responses differing by > 1 point (on the 0–12 scale) triggered a consensus meeting; otherwise, the mean of the three scores was retained. The resulting human consensus served as the reference standard. Inter-rater agreement was excellent (two-way random-effects ICC = 0.84).

### LLM grading procedure

Both ChatGPT-4 (OpenAI, 2025 API) and DeepSeek-3 (2025 public release) were implemented using a zero-shot prompting technique to assess the intrinsic grading capabilities of large language models (LLMs) without requiring external intervention or fine-tuning. Zero-shot prompting was chosen to ensure the models only utilized their pre-trained knowledge, without being exposed to sample answers, calibration examples, or feedback before grading, following recent studies evaluating AI in medical and dental education [6, 22–24].

Each model received the same standardized rubric-based prompt, instructing it to act as an expert dental educator:

“You are an expert dental educator. Please score the following student response using the provided rubric. Evaluate each of the four domains (Clarity, Accuracy, Completeness, and Grammar & Language) from 0 to 3 and provide a total score out of 12. Base your evaluation strictly on the rubric.”

The full text of the scoring rubric was embedded in each prompt to guarantee uniformity and transparency. Models were not provided with previous responses or answers. AI-generated outputs were post-processed to extract scores. In cases where numeric scores were ambiguous, two SME jointly interpreted the AI’s rationale to assign a score.

To maintain the integrity of the zero-shot setting, neither model was given any memory of prior items and was tasked with independently grading each of the 2,358 student responses.

Domain-level and overall scores were extracted from the model outputs using programmatic parsing. Two separate SMEs examined the AI-generated justification to assign the closest suitable score based on rubric alignment in the rare cases where numerical scores were unclear.

### Subgroup coding for stratified analyses

To probe conditions that might influence grading reliability, each response was additionally labelled on four axes:

1. Cognitive level – Remember, Apply/Analyze, Evaluate (per Bloom) [25].
2. Response style – “For secondary analysis, each student response was classified as *Concise*, *Verbose*, or *Flawed* based on criteria developed by the study team. Responses were labeled as:
  Concise: Direct, clear, and relevant, containing only the information needed to answer the question.
  Verbose: Containing redundant, excessive, or off-topic information beyond what was necessary for a complete answer.
  Flawed: Disorganized, lacking coherence, or demonstrating reasoning errors that undermine the answer’s validity.
3. Rubric outcome category (human SMEs’ classification of the accuracy and quality of students’ responses) – Ideal (10–12), Borderline (6–9), Incorrect (0–5) according to human score [26].
4. AI–human error tier (post-hoc) – Low (≤ 1 pt), Moderate (2–3 pts), High (> 3 pts) [27].

### Statistical analysis

All the statistical analyses were conducted by R (version 4. x). Inter-rater reliability with three faculty graders was evaluated by calculating a two-way random effects intraclass correlation coefficient (ICC), which revealed excellent agreement (ICC = 0.84). The 12-point rubric’s internal consistency was examined with Cronbach’s alpha, with an overall α = 0.84 and subscale αs of 0.80 to 0.87. We compared the AI with the human scores by calculating Pearson correlations and paired t-tests to investigate agreement and bias. A score error of each response was calculated, and the responses’ scoring errors were categorized into Low (≤ 1), Moderate (2–3), and High (> 3) tiers. Chi-square tests compared model performance across these tiers, and descriptive statistics summarized exact score differences.

Rubric classifications (Ideal, Borderline, Incorrect) were analyzed using Cohen’s kappa and McNemar’s tests to evaluate misclassification rates and directional bias. We also performed stratified analyses across cognitive levels, response styles, and rubric categories using Kruskal–Wallis tests to detect variations in scoring error.

Lastly, mixed-effects regression models were used to identify predictors of AI–human score differences, with fixed effects for cognitive level, response style, and rubric category, and random intercepts for exam questions. Bonferroni adjustments were applied to control for multiple comparisons.

## Results

### Overall dataset

**Fig. 1:**
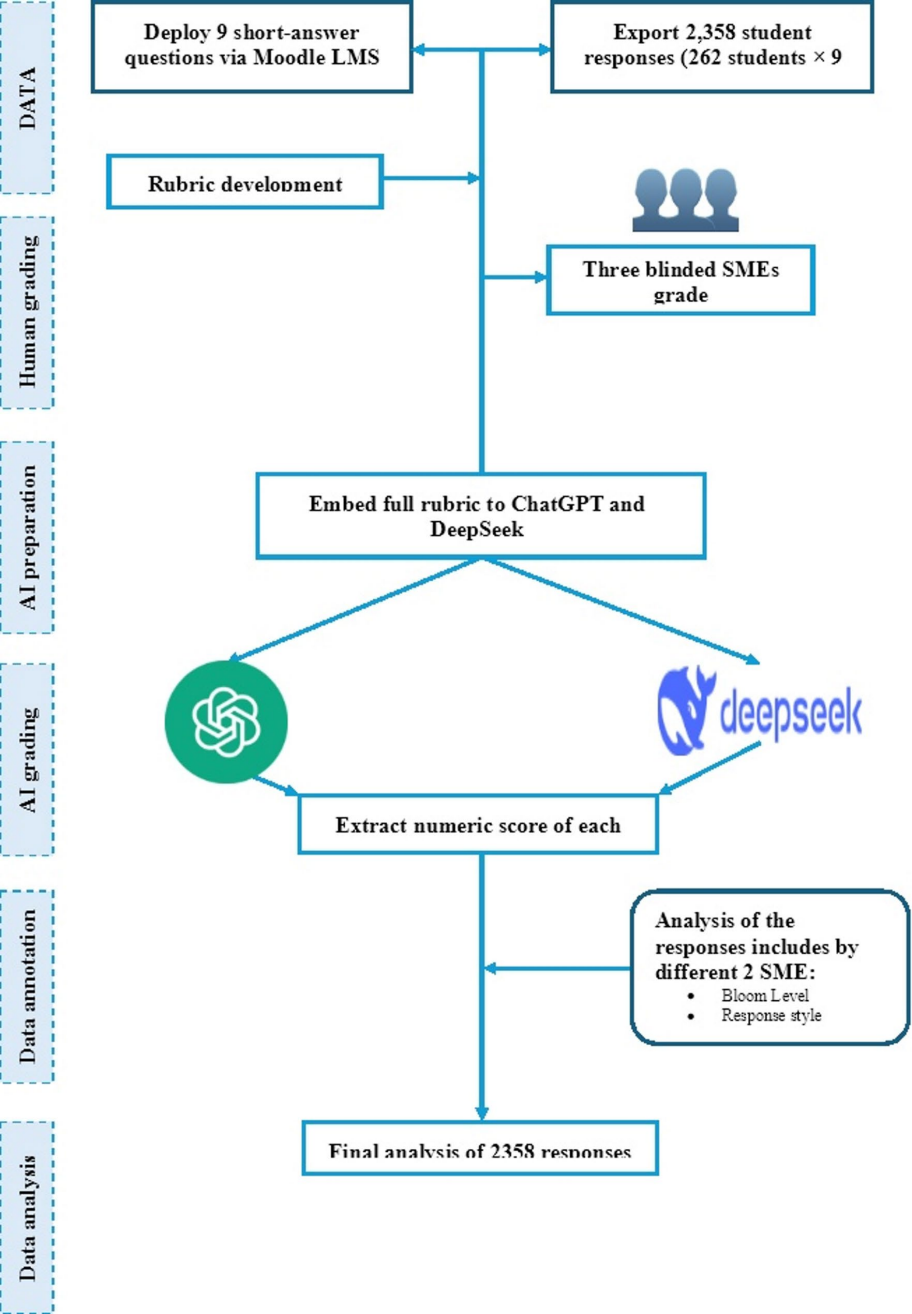
Study design

A total of 262 student responses (9 short-answer questions, *n* = 2,358) were graded by calibrated SMEs using a 12-point rubric, then scored in parallel by ChatGPT-4 and DeepSeek-3 with the embedded rubric; tagged data (Bloom cognitive level, response style, SMEs’ response classification) underwent final agreement and error analyses, as shown in Fig. 1.

### Response characteristics and rubric distribution

**Fig. 2:**
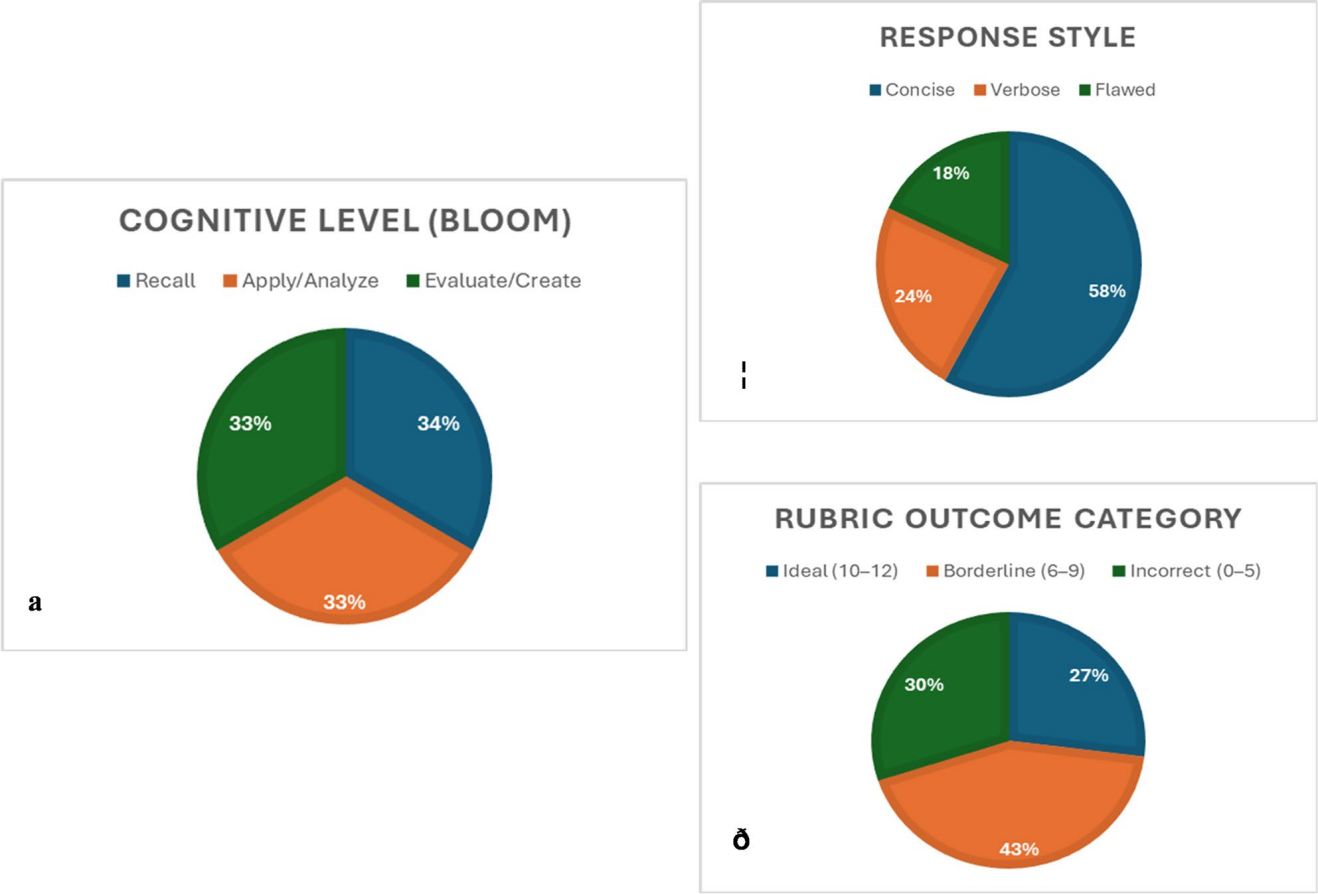
Descriptive breakdown of exam questions and student response characteristics. **a** Cognitive Level Distribution based on Bloom’s taxonomy, showing equal representation of Recall (34%), Apply/Analyze (33%), and Evaluate/Create (33%) questions. **b** Response Style Classification of student answers, with most responses categorized as Concise (58%), followed by Verbose (24%) and Flawed (18%). **c** Rubric Outcome Category based on expert grading, indicating that 27% of responses were rated Ideal (scores 10–12), 43% as Borderline (6–9), and 30% as Incorrect (0–5)

A total of 2,358 short-answer responses were analyzed, evenly distributed across Bloom’s cognitive levels (Recall, Apply/Analyze, Evaluate; 33% each). The majority of student answers were categorized as Concise (58%), with fewer being Verbose (24%) or Flawed (18%). Human SME grading assigned 27% of responses to the Ideal category (scores 10–12), 43% to the Borderline (6–9), and 30% to the Incorrect (0–5), as shown in Fig. 2.

### Model score patterns and reliability

The average human-assigned score was 6.8 ± 3.9. Both models’ mean scores (~ 7.0) were very close to the human graders’ mean of 6.8, although ChatGPT-4’s scores were more variable (SD 4.3) compared with DeepSeek-3’s (SD 3.6). Relative to the human benchmark, DeepSeek-3 obtained an ICC of 0.87, which is considered excellent, while ChatGPT-4 obtained an ICC of 0.64, which is considered moderate according to standard ICC thresholds.

Neither model showed statistically significant overall bias in mean scores (*p* > 0.05). A direct comparison revealed no meaningful score difference between the models (mean difference + 0.03 points, *p* = 0.94) as presented in Table 1.

**Table 1:** Inter-rater agreement and score differences between human and AI graders

Comparison	ICC (2-way, absolute)	Pearson’s *r*	Mean Score Difference (AI – Human)	95% CI for Mean Diff	*p*-value (t-test)
Human vs. ChatGPT-4	0.639	0.64	0.156	−0.575 to 0.886	0.673
Human vs. DeepSeek-3	0.87	0.872	0.183	−0.219 to 0.586	0.368
ChatGPT-4 vs. DeepSeek-3	0.644	0.649	0.028	−0.678 to 0.734	0.938

Mean Score Difference reflects the average (AI – Human) difference in scores out of 12. 95%, *CI* Confidence Interval for the mean difference. *p*-value is from a paired two-tailed t-test comparing AI and Human scores

*ICC* Intraclass Correlation Coefficient (2-way, absolute agreement), *r* Pearson’s correlation coefficient

### Rubric boundary misclassification

At the pass–fail threshold (score 4–7), ChatGPT-4 misclassified 51.5% of responses, while DeepSeek-3 misclassified 33.3%. Agreement with human graders was negligible for ChatGPT-4 (κ = − 0.176) and fair for DeepSeek-3 (κ = 0.284); McNemar p-values were > 0.05 for both models. In the Ideal range (score ≥ 7), flip rates were 25.0% for ChatGPT-4 and 15.9% for DeepSeek-3, with κ = 0.49 and 0.678, respectively, as presented in Tables 2 and 3.

**Table 2:** Rubric category flip-rate and agreement statistics by score band (ChatGPT-4 vs. DeepSeek-3)

Band (Score Range)	Model	Flip Rate (%)	Difference vs. Other Model	Cohen’s κ	McNemar *p*
A (4–7)	ChatGPT-4	51.5%	+ 18.2% vs. DeepSeek	–0.176	0.629
	DeepSeek-3	33.3%		0.284	0.227
B (7–12)	ChatGPT-4	25.0%	+ 9.1% vs. DeepSeek	0.494	1.000
	DeepSeek-3	15.9%		0.678	1.000

Band A includes responses with human scores between 4–7 (near the Incorrect/Borderline threshold); Band B includes scores between 7–12 (near the Borderline/Ideal threshold). Flip Rate is the percentage of responses where the model’s rubric classification disagreed with the human’s. “Difference vs. Other Model” indicates the increase in flip rate relative to the better-performing model. Cohen’s κ measures two-level agreement beyond chance. McNemar p tests for directional bias (*p* < 0.05 = significant asymmetry)

**Table 3:** Confusion matrix comparison: human vs. AI classification accuracy by rubric category

Human Category	AI Model	Correct Classification	Misclassified as Borderline (1-level)	Misclassified as Incorrect (2-level)	2-Level Misclassification Rate	Key Gap (Effect Size) (Correct Classification)
Borderline	ChatGPT-4	498 (48.7%)	236 (23.1%)	288 (28.2%)	28.2%	–20.5% vs. DS
	DeepSeek-3	707 (69.2%)	105 (10.3%)	210 (20.5%)	20.5%	—
Ideal	ChatGPT-4	498 (76.1%)	105 (16.0%)	26 (4.0%)	4.0% (critical misclassification)	–7.9% vs. DS
	DeepSeek-3	550 (84.0%)	79 (12.1%)	0 (0.0%)	0.0% (no critical flips)	—
Incorrect	ChatGPT-4	445 (62.9%)	210 (29.7%)	52 (7.4%) *(over-scored as Ideal)*	7.4% (critical misclassification)	–18.6% vs. DS
	DeepSeek-3	576 (81.5%)	105 (14.8%)	26 (3.7%)	3.7%	—

Two-level misclassifications refer to the most severe rubric errors in which an “Ideal” response is misclassified as “Incorrect” or vice versa. These critical flips can result in unjust penalization or unwarranted credit in summative assessments, and are thus highlighted separately from one-level errors (e.g., Ideal → Borderline or Incorrect → Borderline)

### Absolute error and tiered discrepancies

**Fig. 3:**
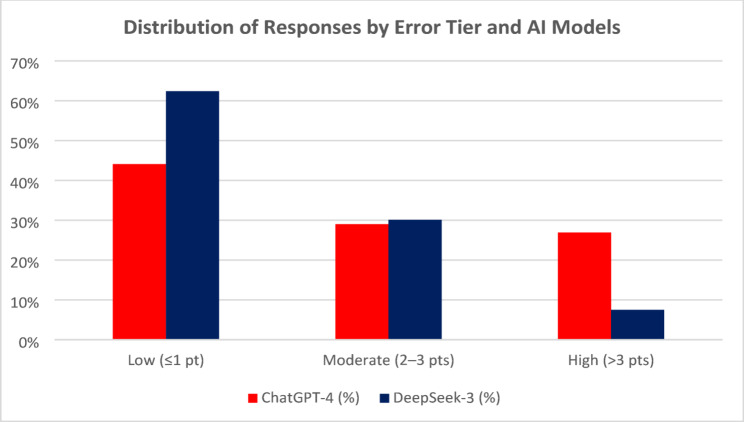
Bar chart comparing the proportion of student responses falling into three absolute error tiers between ChatGPT-4 and DeepSeek-3

DeepSeek-3 reproduced the exact human score in 43.3% of cases and stayed within ± 1 point in 62.4%. ChatGPT-4 achieved this in 35.5% and 44.1% of cases, respectively. High errors (> 3 points) were observed in only 7.5% of DeepSeek-3’s grades versus 26.9% for ChatGPT-4 (p-value = 0.004) as presented in Table 4 and Fig. 3.

**Table 4:** Distribution of absolute score error tiers and mean error magnitude by model

Tier	ChatGPT-4		DeepSeek-3		χ² *p*-value
	*N* (%)	Mean ± SD	*N* (%)	Mean ± SD	
Low (≤ 1 point difference)	1 040 (44.1%)	0.220 ± 0.419	1 472 (62.4%)	0.319 ± 0.463	0.004
Moderate (2–3 points)	684 (29.0%)	2.352 ± 0.617	709 (30.1%)	2.232 ± 0.616	0.004
High (> 3 points)	634 (26.9%)	6.068 ± 2.254	177 (7.5%)	5.875 ± 2.955	0.004

Mean ± SD reflects the average absolute error (± standard deviation) within each tier

χ² *p*-value tests whether the distribution across error tiers differs significantly between models (ChatGPT-4 vs. DeepSeek-3). A value of *p* = 0.004 indicates a statistically significant difference

### Stratified analyses by cognitive level and response style

DeepSeek-3 maintained consistent agreement across all Bloom levels (ICC = 0.876–0.915), showing no significant change in error across cognitive complexity (*p* = 0.214). ChatGPT-4, by contrast, showed significant variability by Bloom level (ICC = 0.537–0.623; *p* = 0.001) as shown in Table 5. When stratified by response style, ChatGPT-4 showed increasing error from Concise (1.70 ± 2.46) to Verbose (2.43 ± 2.26) and Flawed responses (4.00 ± 2.97; *p* = 0.005). DeepSeek-3’s error remained stable across styles (1.02–1.41; *p* = 0.317) as presented in Table 6.

**Table 5:** Inter-Rater reliability and grading consistency across cognitive levels

Level	SME vs. ChatGPT			SME vs. DeepSeek		
	ICC	*r*	KW *p*-value	ICC	*r*	KW *p*-value
Recall	0.537	0.54	0.001	0.876	0.877	0.214
Apply/Analyze	0.623	0.625	0.001	0.912	0.915	0.214
Evaluate/Create	0.589	0.592	0.001	0.901	0.903	0.214

*ICC* Intraclass correlation coefficient (2-way, absolute agreement) between human scores and AI model scores, *r* Pearson correlation coefficient, *KW* *p*-*value* Kruskal–Wallis test *p*-value for differences in absolute error across cognitive levels

**Table 6:** Mean absolute error by response style for ChatGPT-4 and DeepSeek-3

Style	*N* (%)	ChatGPT-4		DeepSeek-3	
		Mean ± SD	KW *P*-Value	Mean ± SD	KW *P*-Value
Concise	1 389 (58.9%)	1.702 ± 2.462	0.005	1.019 ± 1.656	0.317
Verbose	577 (24.5%)	2.432 ± 2.259	0.005	1.409 ± 1.593	0.317
Flawed	392 (16.6%)	4.000 ± 2.966	0.005	1.281 ± 0.999	0.317

Values represent the mean ± standard deviation of absolute error between model and human scores, stratified by response style. Kruskal–Wallis *p*-values (KW *p*) indicate differences in model error across styles. “Flawed” responses yielded significantly higher error for ChatGPT-4 (*p* = 0.005), but not for DeepSeek-3 (*p* = 0.317). Sample size and proportions are identical across models

### Rubric outcome effects

Across rubric bands, ChatGPT-4 was least accurate on Incorrect (2.78 ± 3.35) and Borderline (2.60 ± 2.24) responses, with improved accuracy on Ideal answers (1.23 ± 2.03; *p* = 0.012). DeepSeek-3’s accuracy remained more consistent (0.65–1.56; *p* = 0.521). These differences contributed to more frequent misclassification events in the ChatGPT-4 outputs as presented in Table 7.

**Table 7:** Absolute scoring error by rubric outcome category for ChatGPT-4 and DeepSeek-3

Category	*N* (*n* %)	ChatGPT-4		DeepSeek-3	
		Mean ± SD	KW *p*-value	Mean ± SD DS	KW *p*-value
Incorrect	710 (30.1%)	2.778 ± 3.346	0.012	1.556 ± 2.272	0.521
Borderline	1 014 (43.0%)	2.603 ± 2.240	0.012	1.205 ± 1.068	0.521
Ideal	634 (26.9%)	1.229 ± 2.027	0.012	0.646 ± 0.992	0.521

Category definitions: Incorrect = scores 0–5, Borderline = 6–9, Ideal = 10–12 (based on human-assigned scores)

*Mean ± SD* Mean absolute error (AI score − Human score) and standard deviation within each rubric outcome category, *KW* *p*-*value* *p*-value from Kruskal–Wallis test comparing absolute errors across rubric categories (within each model separately)

### Mixed-effects modeling of AI–human score discrepancy

#### Mixed-effects model for ChatGPT-4

Regarding the mixed-effects model examining ChatGPT-4’s scoring behavior relative to human graders, Table 8 summarizes coefficient estimates for several predictors, using the difference in scores (ChatGPT-4 – Human) as the outcome. The reference condition is defined as a Borderline response with Concise style at the Apply/Analyze cognitive level. None of the cognitive level or response style predictors reached statistical significance after Bonferroni adjustment, indicating that ChatGPT-4’s scoring discrepancies were not meaningfully influenced by question type or student response length/clarity.

**Table 8:** Mixed-effects regression coefficients: ChatGPT-4 score minus human score, with Bonferroni-Adjusted significance

Predictor	Coefficient Estimate	Standard Error	Wald z-statistic	*P*-value	Bonferroni-adjusted *p*-value	95% CI	
						Lower	Upper
Intercept	−0.309	1.334	−0.232	0.817	1	−2.924	2.306
Cognitive Level = Evaluate/Create	1.679	1.734	0.968	0.333	1	−1.720	5.078
Cognitive Level = Remember	−0.239	1.759	−0.136	0.892	1	−3.686	3.208
Response Style = Flawed	0.642	0.908	0.707	0.480	1	−1.139	2.422
Response Style = Verbose	−0.914	0.803	−1.139	0.255	1	−2.487	0.659
Rubric Outcome Category = Ideal (10–12)	−1.445	0.772	−1.872	0.061	0.366	−2.959	0.068
Rubric Outcome Category = Incorrect (0–5)	1.599	0.735	2.176	0.030	0.18	0.159	3.038

The model includes fixed effects for cognitive level, response style, and rubric outcome category, with a random intercept for each question (*n* = 2358 responses, 9 question groups). The baseline (intercept) corresponds to a Borderline response, written concisely, at the Apply/Analyze cognitive level. Bonferroni-adjusted *p*-values correct for multiple comparisons across six predictors (α-adjusted = 0.05/6 ≈ 0.0083). Only the Incorrect category remained significant after adjustment (*p* = 0.03; adjusted *p* = 0.18)

#### Influence of rubric category on ChatGPT-4 scoring

**Fig. 4:**
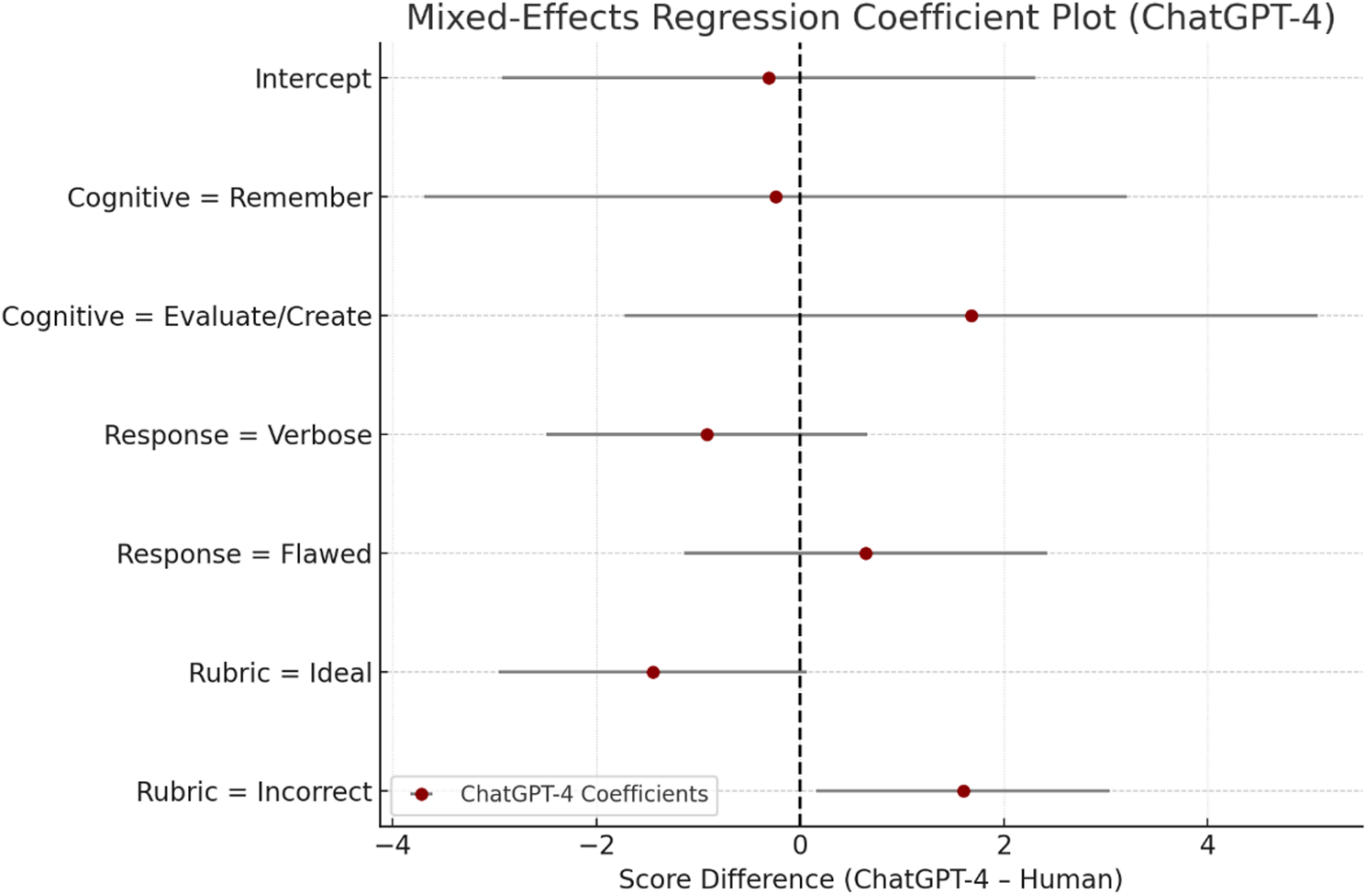
Mixed-effects regression coefficient plot for ChatGPT-4 score differences relative to human grading. Dots represent coefficient estimates with 95% confidence intervals. Positive values indicate over-scoring by ChatGPT-4. The model significantly over-scored responses labeled “Incorrect,” with no other predictors reaching statistical significance after adjustment

The rubric outcome category showed the strongest directional effects on ChatGPT-4’s scoring behavior **(**Table 8**)**. Responses that were Incorrect according to human graders tended to be over-scored by ChatGPT-4 (β = +1.599, *p* = 0.030), although this effect did not remain significant after Bonferroni correction (adjusted *p* = 0.18). In contrast, Ideal responses were slightly under-scored (β = −1.445, *p* = 0.061; adjusted *p* = 0.366). While these effects did not reach adjusted significance, the pattern suggests a tendency to over-credit weaker answers and modestly under-credit stronger answers. The model intercept (β = −0.309, *p* = 0.817) indicates no systematic bias under the reference condition. These trends are illustrated in Table 8 and Fig. 4.

#### Mixed-effects model for DeepSeek-3

For all of the mixed-effects model predicting score differences between DeepSeek-3 and human graders, the following patterns were observed. The intercept (0.245, *p* = 0.668) captures the average DeepSeek–Human score difference for the reference group (Apply/Analyze level, Concise response, Borderline rubric category) and is not significant.

In terms of cognitive level, neither “Evaluate/Create” (− 0.635, *p* = 0.342) nor “Remember” (0.181, *p* = 0.793) showed a significant effect, indicating that DeepSeek’s deviation from human scores did not systematically vary by question complexity. Similarly, Verbose response style was not associated with a significant score shift (0.313, *p* = 0.517).

**Fig. 5:**
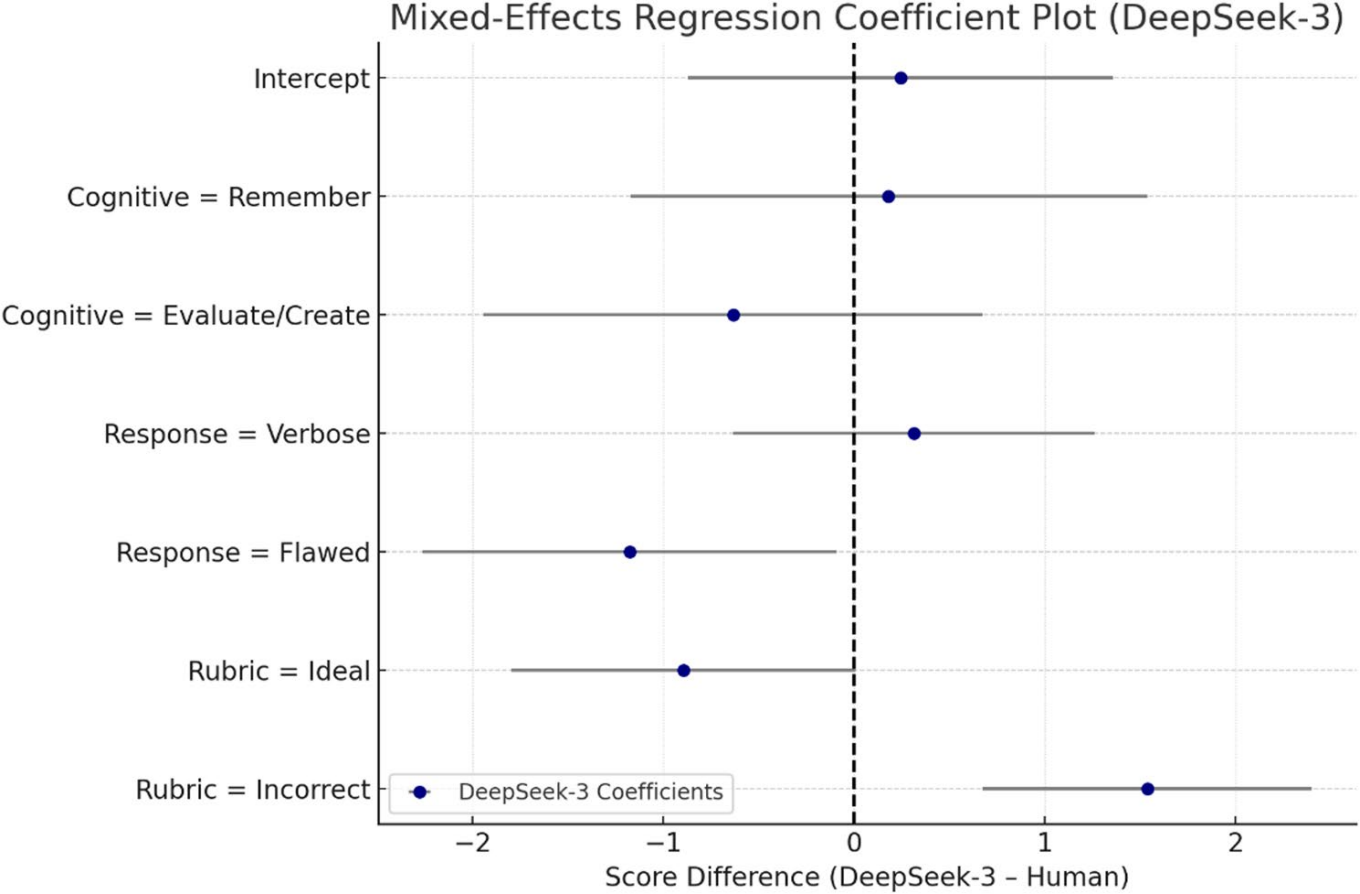
Mixed-effects regression coefficient plot for DeepSeek-3 score differences relative to human grading. Dots represent coefficient estimates for each predictor; horizontal bars indicate 95% confidence intervals. Positive values indicate over-scoring by DeepSeek-3. The rubric category “Incorrect” showed significant over-scoring, while other effects were non-significant after correction

However, two predictors reached statistical significance. First, Flawed response style had a significant negative coefficient (− 1.176, *p* = 0.034; Bonferroni-adjusted *p* = 0.204), suggesting DeepSeek-3 tended to assign scores approximately 1.18 points lower than human graders for disorganized or poorly written responses, though this effect does not survive strict multiple comparison correction. Second, and more importantly, the Incorrect rubric category demonstrated a strong and statistically significant positive effect (1.537, *p* < 0.001; Bonferroni-adjusted *p* < 0.006), suggesting that DeepSeek was providing over-gradable incorrect responses over 1.5 points relative to the human benchmark, as shown in Table 9 and Fig. 5.

**Table 9:** Mixed-effects regression coefficients: DeepSeek-4 score minus human score, with Bonferroni-Adjusted significance

Predictor	Coefficient Estimate	Standard Error	Wald z-statistic	*P*-value	Bonferroni-adjusted *p*-value	95% CI	
						Lower	Upper
Intercept	0.245	0.570	0.429	0.668	1	−0.872	1.361
Cognitive Level = Evaluate/Create	−0.635	0.668	−0.950	0.342	1	−1.945	0.675
Cognitive Level = Remember	0.181	0.692	0.262	0.793	1	−1.174	1.537
Response Style = Flawed	−1.176	0.554	−2.124	0.034	0.204	−2.262	−0.091
Response Style = Verbose	0.313	0.483	0.649	0.517	1	−0.634	1.260
Rubric Outcome Category = Ideal (10–12)	−0.895	0.461	−1.943	0.052	0.312	−1.799	0.008
Rubric Outcome Category = Incorrect (0–5)	1.537	0.440	3.491	< 0.001	< 0.006	0.674	2.400

The model includes fixed effects for cognitive level, response style, and rubric outcome category, with a random intercept for each question (n = 2358 responses, 9 question groups). The baseline (intercept) corresponds to a Borderline response, written concisely, at the Apply/Analyze cognitive level. Bonferroni-adjusted *p*-values correct for multiple comparisons across six predictors (α-adjusted = 0.05/6 ≈ 0.0083). Only the Incorrect category remained significant after adjustment (*p* = 0.03; adjusted *p* = 0.18)

The Ideal category is moving to align with human scoring (− 0.895, *p* = 0.052), so it would be expected to under-score ideal answers slightly; however, the effect is not significant post-correction. In this dataset, it was found that the most consistent error pattern exhibited by DeepSeek-3 was over-crediting wrong answers, and performance seems to be similar across question types as well as less dependent on verbosity.

## Discussion

The use of LLMs in educational assessment is increasingly being discussed as a means for economies of grading scale, but also for providing prompt, scalable feedback in higher education fields. Yet, concerns remain about the reliability, validity, and fairness of these models, especially in high-stakes contexts such as clinical dental education, in which refined judgment and rubric adherence are necessary [1, 6]. Although commercial models, such as ChatGPT-4, have promising utility for formative assessments, their consistency with human graders varies greatly across tasks, response types, and instructional domain [2].

Recent analyses have pointed out ChatGPT-4’s bias toward “optimism”, its “rubric drift,” and its inconsistency in scoring at cognitive boundaries [28], and have warned against an uncontrolled deployment of the model in high-stakes testing. In this context, our study makes an original comparison between a commercial LLM (ChatGPT-4) and an open-weight rival (DeepSeek-3), based on rubric-based short-answer grading in undergraduate dental education. This work is particularly important at the moment; institutions of higher education are now integrating AI-enabled grading workflows, raising the bar on both psychometric standards and practical feasibility.

This cross-sectional study provided important insights into the potential of large-language models (LLMs) for automating the grading of clinical short-answer responses in dental education. Given the growing interest in scalable assessment solutions, our comparison of ChatGPT-4 and DeepSeek-3 against expert human graders addresses a critical gap in current knowledge.

Our work contributes to an emerging literature on the role of LLMs in dentistry. Previous works have effectively compared the performance of chatbots on standard dental exams and ascertained the quality of their responses to clinical queries in the areas of pediatric dentistry, special needs care, and prosthodontics [17–20]. The previous works concluded that LLMs hold immense foundational knowledge. But our study shifts the focus from evaluating the AI as a student-taker to using it as an evaluator. The demonstrated reliability of DeepSeek-3 in marking actual students’ answers draws on prior accounts of AI capability and brings the use of LLMs into the real-world, high-volume realm of academic testing, the bridge to operational integration into dental programs that is required.

Our preliminary observations on inter-grader reliability and score bias reveal stand-out differences between ChatGPT‑4 and DeepSeek‑3 compared to a previous study that demonstrated ChatGPT’s lower correlation with human scorers than inter-grader human reliability in chemistry SAQ grading (*r* ≈ 0.56 versus 0.75) [29].

DeepSeek-3’s consistency was particularly noticeable, a positive characteristic indicating that it could be a dependable supplement for dental training. In addition to reliability indices, the size and pattern of grading errors appeared as important distinguishing features between models. DeepSeek-3 showed less variability and produced far fewer high-stakes (> 3-point) errors, whereas ChatGPT-4 was more variable and much less efficient at controlling large errors. Misunderstanding around critical rubric thresholds remained an issue for both automated systems, but DeepSeek-3 nevertheless reduced the risk of high-stakes misclassification compared with ChatGPT-4. This discrepancy underlines the importance of model selection and the continued need for human oversight.

An analysis of cognitive complexity will advance our understanding of the strengths and weaknesses of each model. The constancy of DeepSeek-3’s performance throughout the levels of Bloom’s taxonomy highlights its robustness against the relative sensitivity of ChatGPT-4 to higher cognitive demand. Another factor affecting model performance was the length and clarity of the response. ChatGPT-4’s vulnerability to inflated errors in verbose or disorganized responses is quite the opposite of DeepSeek-3’s robustness. This finding has practical applications when parameters in the LLM-based grading system are to be set in different education settings.

The superior performance of DeepSeek-3 over ChatGPT-4 is in line with our hypothesis and due to the different architecture for how the models were trained. ChatGPT-4 is pre-trained for general dialogue and user-focused fluency, while DeepSeek-3’s architecture might favor more technical or structured corpora, which is conducive to following rubric logic more closely. Stable performance of the AI scores was observed at different Bloom’s levels and response styles, indicating that DeepSeek-3’s generalization capability for academic assessment tasks is better. We take our results as evidence of the utility of task-aligned LLMs and evidence-based calls for clear benchmarking in educational deployment.

 Regarding overall reliability and score bias, DeepSeek-3 achieved excellent reliability (ICC = 0.87), exceeding the *strong* ICC range ChatGPT-4 has shown in essay-based dental assessments (0.79–0.86) [30] and reported for ChatGPT-4 in prior short-answer studies (κ ≈ 0.63 in an undergraduate medical course [2]; ICC ≈ 0.70 in a dental essay examination [6]. It’s near-zero mean bias (+ 0.18 pts) also contrasts with GPT-4’s systematic under-scoring in multi-course medical datasets (median − 0.41 pts) [6] and ChatGPT-4o’s 1.34/10-point bias in a postgraduate ventilation course [31]. Collectively, these comparisons confirm that DeepSeek-3 meets and, in some contexts, exceeds the upper reliability range so far documented for commercial LLMs, whereas our ChatGPT-4 ICC = 0.64 aligns closely with earlier reports.

 While regarding error magnitude and distribution, our observation that ChatGPT-4 produced high-impact discrepancies (> 3 pts) in 269% of answers parallels the 0% “out-of-tolerance” rate seen in Jade & Yartsev’s postgraduate study [31] and the broad variance described by Shermis, who found large QWK swings across prompts when ChatGPT was benchmarked against secondary-school essays [14]. By contrast, DeepSeek-3s 7.5% large-error rate is markedly lower than even the most favorable GPT-4 estimats (≤ 15%) in the 12-course medical evaluation by Grévisse et al. [6]., underscoring its tighter error bands.

Although the grading reliability of LLMs in dental education was the main focus of our study, new research in related fields provides convergent evidence on model performance variation [32]. In their evaluation of DeepSeek-V3 and ChatGPT-4o for patient education, for example, Dincer and Dogu (2025) [32] discovered that DeepSeek offered more precise and contextually relevant answers to frequently asked questions about laparoscopic surgery. Their findings also highlight the benefits of open-weight models like DeepSeek in domain-specific applications that demand high factual precision, despite the task being different from rubric-based grading. In conjunction with our results, this implies that training alignment and model architecture might be important factors in downstream performance on clinical and educational communication tasks.

Misclassification of borderline scripts is a recurring weakness of AI graders. Shermis (2024) highlighted error inflation around cut-scores in national writing samples [14], and Grévisse reported similar drift for GPT-4 despite otherwise “acceptable” reliability [6]. Our DeepSeek-3 reduced pass–fail flips to33%, whereas ChatGPT-4 mis-labelled one in two scripts, mirroring te 52% flip rate that prompted Li et al. to advocate a human-in-the-loop framework (GradeHITL) for rubric moderation [33]. Hence, our data reinforce the consensus that fully automated summative use remains unsafe, but they also show that model choice substantially alters the risk profile.

Regarding cognitive level stratification, DeepSeek-3 kept ICC values > 0.87 across Recall, Apply/Analyze, and Evaluate questions, supporting the claim from Grévisse that LLMs can grade “high-quality keys” consistently, independent of cognitive load [6]. Conversely, ChatGPT-4’s reliability dipped on higher-order items, echoing Jade & Yartsev’s finding that evaluative questions suffered the poorest agreement (ICC = 0.09) [6]. Poličar’s bioinformatics course experiment likewise flagged widening LLM-human gaps as item complexity rose [34], suggesting our stratified approach adds convergent validity across disciplines.

While regarding the influence of response style, we found ChatGPT-4’s absolute error more than doubled on verbose or poorly structured prose, a vulnerability that Shermis attributed to the model’s length-based heuristics [14]. DeepSeek-3, by contrast, showed style-invariant performance, dovetailing with Grevisse’s report of only a “weak correlation” between answer length and GPT-4 error, and extending it by demonstrating true robustness in an open-weight model [6].

In terms of respect to propensity to over-score incorrect answers, mixed-effects modelling revealed that both LLMs, but especially DeepSeek-3 (+ 1.54 pts), over-credited incorrect responses. This “false-positive generosity” mirrors the pattern catalogued in Bisante et al.’s HCI audit of LLM mis-judgements, which advised explicit down-weighting rules to curb user-facing risk [27]. Similar inflation was observed in Poličar’s live-classroom deployment, where ChatGPT leniently marked lower-quality bioinformatics answers [34]. Our data, therefore, confirm that optimism bias persists even in technically superior models and must be explicitly mitigated.

Finally, our results reinforce the central insight from Li et al.’s GradeHITL framework that algorithmic gains do not obviate human oversight but can strategically focus faculty attention on the residual 8–27% of “high-tier” discrepancies [33]. The markedly lower variance and misclassification rates achieved by DeepSeek-3 show tangible progress relative to the GPT-4 literature, yet they also illustrate why safety-critical deployment in dental education should remain human-moderated until the systematic over-scoring of incorrect work is solved.

In sum, every quantitative advantage or shortcoming we observed aligns with or extends specific peer-reviewed findings, confirming the external validity of our dataset and underscoring the need for model-specific governance rather than one-size-fits-all-policies.

Differences in grading reliability between ChatGPT-4 and DeepSeek-3 likely reflect core distinctions in model architecture, data exposure, and alignment to structured scoring criteria. LLMs like ChatGPT-4, which are trained on a large and diverse collection of text, may become oversensitive to input ambiguity, especially when encountering variable student prose or weak responses. This “optimism bias” and propensity to generate interpretations of intent from partial or incomplete responses is well-documented in LLM audit investigations and may account for the model’s over-crediting of incorrect or flawed answers and potential over-estimation of the rate of borderline misclassification and false negative rate estimated for passing examinees with sub-minimal competency [27].

Mechanism-wise, the preference of DeepSeek-3 for the cognitive levels and response styles may come from its architecture and/or the experience (training process), where the latter may not only include plain text, comprising instructional or technical language, with the logic and reasoning of the rubric being more uniform. So, this would, in consequence, favor the rather “conservative” scoring, narrow error bands, and better generalization across question complexity, as was generally found in recent LLM comparative studies [6, 34].

The critical challenge for both models arises at the boundary of rubric categories, especially for “borderline” answers, where prior research confirms LLMs are most likely to diverge from human expert judgment [14, 33]. In clinical dental education, these are precisely the scripts where patient safety and graduation standards depend on high-fidelity discrimination between minimal and unacceptable competence. AI models, if left unchecked, could propagate errors that undermine assessment validity, as corroborated by multi-cohort grading experiments and recent safety audits [6, 33].

From an educational perspective, the results of this study support the call for ongoing human supervision, particularly in situations of high jeopardy. Automated scoring can allow formative assessment at scale, but systematic calibration, error flagging, and moderation by faculty need to remain embedded in deployment to guard against AI’s bias towards “filling in gaps” and rewarding partial answers. Only through such governance can LLMs safely augment rather than replace expert grading in health professions education.

Our findings indicate that integrating LLMs, particularly open-weight models like DeepSeek-3, into routine grading workflows can substantially reduce faculty workload without sacrificing reliability. A tiered approach, where AI provides first-pass scores and human experts review only responses with > 1-point AI–human divergence, could cut marking time by up to 50–60% while maintaining psychometric rigor. For clinical educators, this means more capacity for individualized feedback, remediation, and curriculum development rather than mechanical scoring.

The policy implications are that dental schools and accreditors may want to provide some direction on the use of AI-supported assessments. Such policies could require standardized prompts, rubric-embedding protocols, and transparent accountability mechanisms to reduce human bias and over-scoring of incorrect answers as a systematic bias in both ChatGPT-4 and DeepSeek-3. Organizations with data-sovereignty requirements can especially appreciate locally deployable open-weight models coalition with General Data Protection Regulation (GDPR) style requirements in Europe and nascent data-privacy laws in the Middle East. In the final analysis, thoughtful use of LLMs can promote equitable, timely feedback in dental education, provided these applications are supported by policy frameworks that mandate human-in-the-loop validation, frequent bias audits, and continued performance monitoring.

Interestingly, ChatGPT-4’s grading errors were disproportionately larger for verbose responses, its variance rising markedly compared to concise answers, suggesting an undue influence of token-length heuristics on its rubric application. In contrast, DeepSeek-3 demonstrated remarkably consistent agreement across all Bloom’s cognitive levels, including Evaluation and Synthesis tasks, indicating that its reasoning capabilities generalize uniformly and defy the anticipated drop in accuracy on higher-order questions.

Although DeepSeek-3 was more reliable in this study, its performance is not without important trade-offs requiring caution. Most troubling, our mixed-effects model found that DeepSeek-3’s most consistent error was a substantial over-scoring of incorrect answers, a severe flaw in a high-stakes clinical educational environment. This “optimism bias,” shared by ChatGPT-4, was both stronger and statistically more consistent in DeepSeek-3. Furthermore, practical implications of its open-weight nature and potential on-premises deployment are a lack of the user-friendly, polished interface and extensive safety filters of a commercial product like ChatGPT-4.

This places more burden on the institution for control of the infrastructure, security, and output consistency of the model. Therefore, the choice between models is not straightforward; it involves trading off ChatGPT-4’s higher variance and responsiveness to response style against DeepSeek-3’s more consistent but systematically lenient scoring of incorrect answers.

### Limitations

There are numerous limitations to this study. Its generalizability may be limited since it was carried out at a single institution in a single cultural and linguistic context (Egypt), with assessments in English. The results show baseline model performance rather than optimized performance as we only used zero-shot prompts and did not test prompt variations or fine-tuning. Furthermore, in a single dental course, we concentrated on short-answer questions; different question types or subjects may produce different results. Despite a large sample, these factors should be considered when interpreting the results.

Two subject-matter experts were needed to adjudicate ambiguous AI outputs in a small percentage of cases, which may have introduced some human bias. Furthermore, one of the models that was evaluated (ChatGPT-4) is proprietary, which means that the complete details of its architecture and training data are not available. This could limit the models’ interpretability and reproducibility. The analytical rubric’s validity outside of the study context was not externally tested, despite the fact that it showed good internal consistency. Finally, performance seen with the 2025 model versions might not be precisely replicated in subsequent iterations due to the periodic updating of LLMs.

To address these limitations, future research efforts are needed, including focused fine-tuning and prompt engineering to improve the grading accuracy [35], longitudinal studies to evaluate long-term reliability, strict human-in-the-loop validation efforts to curb errors, cross-cultural and multilingual evaluations to ensure equitable treatment, and extensive cost-benefit analyses to inform educational technology policy.

Our results should be most generally relevant to other dental schools with similar English-medium curricula, and extrapolation to other disciplines (e.g., prosthodontics) or to non-English settings should perhaps be exercised with caution, as institution and faculty-based elements such as curriculum design and English-language proficiency have ramifications for the performance of AIs when grading. From a technological perspective, while DeepSeek-3’s open-weight license enables on-premise deployment necessary in regions with stringent data sovereignty laws, cloud-based GPT-4 may suffer from latency and cost limitations in resource-constrained settings. For further validation, we recommend that large, multicenter studies in different cultural settings be conducted that would include validation against AI grading in alternative assessment formats, including case reports and the Objective Structured Clinical Examination (OSCE) reflection notes, and fine-tuning experiments with the objective to balance incremental accuracy gains with privacy trade-offs.

Although our study used a zero-shot method by design as a baseline, the error pattern observed, particularly the systematic over-scoring of incorrect answers, presents clear paths to improvement. Prompt engineering offers a cheap, short-term route for improvement [10]. For instance, a few-shot prompting, where multiple exemplar answers for each score range are provided to the model, potentially has the capacity to get its scoring more in line with the rubric [35].

Similarly, chain-of-thought prompting can also be used to elicit the model to reason stepwise (e.g., “First evaluate the answer for Clarity and provide a reason, then for Accuracy.“) before aggregating these into a final score, thereby reducing arbitrary choices and increasing transparency. To produce more substantial gains, tuning on a hand-curated collection of human-graded dental responses would explicitly tune the parameters of the model towards the domain-related linguistic conventions, clinical reasoning, and rubric usage required here [35, 36]. This would especially reverse the noted optimism bias by training the model to severely penalize factual mistakes and faulty logic, even in otherwise satisfactory sentences. A blended strategy, namely a well-crafted prompt along with limited fine-tuning, is an appealing path to realizing the high reliability required for summative assessment.

### Practical implications for dental education

Our results show that DeepSeek-3, an open-weight LLM, reduces faculty workload while preserving assessment integrity by achieving grading reliability comparable to human experts (ICC = 0.87). In dental education, where sizable cohorts produce thousands of short-answer responses that demand prompt feedback, this has a particularly significant impact. Institutions can redirect faculty resources toward clinical training and individualized instruction—two crucial areas highlighted in recent dental education reforms—by automating rubric-based scoring [6, 9].

For successful deployment, dental schools must adopt a strategic approach focused on three pillars:

1. Strategic Selection of Cost-Efficient Model: Open-weight nature of DeepSeek-3 offers a better long-term value proposition. Compared to API-based models like ChatGPT-4, having unsteady, periodic costs, DeepSeek-3 involves a fixed one-time cost in on-premises or cloud infrastructure, being more economical for scaling through large classes and across multiple courses.
2. Seamless Integration for Scalability: To realize actual efficiency dividends, AI grading must be integrated with what already works. This means developing a computerized pipeline that can interact with the institutional Learning Management System (LMS) to batch-export student responses, process them through the LLM with the embedded rubric, and spit out scores to the gradebook without labor-intensive human error.
3. A Phased, Human-in-the-Loop implementation to minimize risk:
  Phase 1 (Pilot): Begin with low-stakes formative assessment to build trust and refine the technical process.
  Phase 2 (Hybrid Grading): Implement for first-pass summative grading. Faculty review is selectively aimed at responses flagged by the system, specifically those with a significant AI-human disagreement (> 1 point), borderline grades, and an independent random sample. This model has the potential to reduce faculty grading time by an estimated 50–70% while upholding academic integrity.
  Critical Safeguard: To counter the identified “optimism bias,” have a mandatory human review of all low-score responses that the AI leniently marks to avoid passing off failing responses inappropriately.

With this streamlined process, dental schools can leverage AI to gain substantial workload savings without compromising assessment integrity, scalability, and cost control.

### Ethical and educational challenges of using AI in assisting grading

The wider educational and ethical ramifications of AI-assisted grading should be carefully considered, in addition to rubric alignment and scoring accuracy. If students perceive that responses are assessed more for rubric compliance than for depth of reasoning, an over-reliance on automated systems may unintentionally de-emphasize reflective writing and critical thinking.

Furthermore, students’ confidence in the assessment process may be weakened by opaque model behavior and inexplicable scores, especially if the feedback is not clear or useful. These dangers show that human supervision is necessary for both psychometric quality and the preservation of clinical education’s educational principles, which include encouraging ethical judgment, contextual awareness, and nuanced reasoning.

### Broader ethical and equity considerations

Aside from near-term issues of reliability and fairness in scoring, introducing AI into learning measurement raises deep ethical and equity concerns that need careful reflection. Equity of access is the first big issue. Employing advanced LLMs, in the form of a subscription service API like ChatGPT-4 or as installed software like DeepSeek-3, requires significant institutional expenditures for licensing, computing equipment, and technical maintenance. This may expand existing gaps between resourced and under-resourced dental schools and produce a “digital divide” in the quality of instruction [37].

Second, and most significantly, is the algorithmic bias and fairness of grading. Our study, not having been designed to detect demographic bias, was in one linguistic and cultural context. It is amply demonstrated that LLMs can behave differently when processing language from non-native speakers or conveying diverse cultural patterns of communication [37, 38]. It is possible for a model to unfairly sanction a correctly accurate response, but one formulated in a style different from the “standard” academic English in which it was predominantly trained. Moreover, implicit bias in the models’ training data on gender, race, or nationality can inadvertently impact clinical scenario assessment, even in short-answer. For instance, a model can associate presentation of disease or treatment options with specific demographic traits and create skewed judgments of student answers, contradicting or confirming such associations.

Therefore, before broad adoption, it is essential to conduct robust fairness audits across diverse student populations. This includes performance difference testing by home language, cultural background, and other demographic categories. Model developers’ openness regarding training data and anticipatory measures towards preventing bias are called for. Ultimately, the purpose of AI integration should be to augment educational justice by providing standardized, timely feedback, but unless closely monitored, it is likely to solidify already present inequities and introduce new, uninterpretable forms of discrimination into high-stakes testing.

### Integration into dental curricula

Curriculum alignment is critical to the success of AI-assisted grading. We suggest three stages for incorporating LLMs into dental education:

1. Formative Assessments: For low-stakes tests that offer immediate feedback, use DeepSeek-3.
2. Summative Exams: Use LLMs as first-pass graders, and have faculty members audit 10–20% of the responses, paying particular attention to pass/fail boundaries and other thresholds.
3. Faculty Training: Workshops to reduce over-scoring bias (found in both models) and adjust rubrics for LLM compatibility.

This gradual implementation is in line with the Association for Dental Education in Europe’s recommendation that assessments strike a balance between feasibility and authenticity [9].

## Conclusions

Across the set of clinical short-answer responses in the dental education program examined in this single-institution dataset, DeepSeek-3, an open-weight LLM, demonstrated better grading agreement with calibrated human experts than ChatGPT-4. DeepSeek-3 also had overall grading concordance with trained human examiners that was better than ChatGPT-4, with lower high-magnitude error and more consistent performance across cognitive levels and response styles. This increased consistency has one specific risk of its own, however: a strong tendency to over-score incorrect answers. Therefore, although DeepSeek-3 has great potential to reduce workload, its deployment needs strong safeguards, specifically ones aimed at flagging and re-scoring low-scoring responses, to mitigate its systematic optimism bias.

However, both models tend to over-score incorrect answers, which necessitates human oversight in high-stakes assessments. Cautiously applied, LLMs such as DeepSeek-3 could aid in reducing the grading workload by humans while keeping educational validity.

Further research is necessary to investigate systematic error patterns, explore the effects of fine-tuning, and assess wider applicability across various courses, institutions, and languages to ensure effective and safe integrative tools in dental curricula.

## Supplementary Information

Supplementary Material 1.

## Data Availability

Research data supporting this publication is available from the corresponding author upon request.
