# Supplementary material for: Calibration of AI large language models with human subject matter experts for grading of clinical short-answer responses in dental education
Source: BMC Oral Health. 2026 Feb 6;26:286. doi: 10.1186/s12903-026-07665-4 (PMC12896245; doi:10.1186/s12903-026-07665-4)
Supplement: Supplementary file 1 — Supplementary Material 1. [file 12903_2026_7665_MOESM1_ESM.docx]

**Supplementary file 1. Periodontology Assessment Items with Stems, Cognitive Levels, and Clinical Scenarios**

| **Q No.** | **Cognitive Level** | **Stem & Question** |
| --- | --- | --- |
| **1** | **Remember** | **Stem:** Oral biofilms play a dual role in oral health and disease. **Question:** Describe the main functions of oral biofilm in both health and disease. |
| **2** | **Remember** | **Stem:** A 25-year-old female is scheduled for root coverage on tooth #13, with a thin gingival phenotype and keratinized tissue width of 1 mm. She is otherwise healthy. **Question:** List two soft tissue–related factors that may influence the predictability of root coverage in this patient. |
| **3** | **Remember** | **Stem:** Inflammatory mediators are key drivers of periodontal tissue destruction. **Question:** List the functions of four key inflammatory mediators involved in periodontal disease and their role in disease progression. |
| **4** | **Apply** | **Stem:** Chronic periodontal disease has been linked to adverse pregnancy outcomes. **Question**: Using current scientific evidence, outline the biological mechanism by which chronic periodontal diseases may contribute to the risk of premature birth. |
| **5** | **Apply** | **Stem:** A 37-year-old male presents with 2 mm gingival recession on tooth #43, with 2 mm of remaining keratinized gingiva, no interproximal attachment loss, good oral hygiene, and no systemic health issues. **Question:** Classify the recession using both Miller’s (1985) and Cairo’s (2011) classification systems. |
| **6** | **Apply** | **Stem:** Not all periodontal defects are suitable for regenerative therapy. **Question:** List five key clinical and radiographic criteria for determining if a periodontal defect is suitable for regenerative therapy. |
| **7** | **Evaluate** | **Stem:** A 30-year-old female presents with 3 mm labial gingival recession on tooth #31, probing depth 1 mm, thin phenotype, 2 mm keratinized tissue, no interproximal loss. She reports hypersensitivity and esthetic concerns, maintains excellent oral hygiene, and has no systemic risks. **Question:** Outline a complete treatment sequence for this patient using minimally invasive periodontal techniques, justifying each step for optimal esthetic and long-term outcomes. |
| **8** | **Evaluate** | **Stem:** 42-year-old male presents with a narrow, deep, three-wall intrabony defect on the mesial aspect of tooth #12, thin gingival phenotype, intact papilla, non-smoker, controlled hypertension. He requests a surgical option with minimal postoperative discomfort and downtime. **Question:** Choose the most appropriate initial surgical technique and explain why it is indicated over alternatives. |
| **9** | **Evaluate** | **Stem:** A 55-year-old male presents with a 7 mm probing pocket depth and a 2-wall intrabony defect on tooth #36. The tooth has Grade II mobility, uncontrolled type 2 diabetes (HbA1c 9.2%), poor oral hygiene, and Class II furcation involvement. Radiographs confirm the vertical defect morphology. **Question:** Evaluate the suitability of regenerative periodontal therapy for this patient, integrating defect morphology, tooth mobility, and systemic health. |
